# Accelerating ambient AI scribe enterprise-scale deployment: Cleveland Clinic’s novel approach to health system-industry partnership

**DOI:** 10.1038/s44401-026-00144-6

**Published:** 2026-08-11

**Authors:** Amy Merlino, Abigail Blue, Eric Boose, Beth Meese, Jennifer Owens, Jean Vickers, Alisa Angeloff, Monica Arnold, RuiJun Chen, Madeline Grade, Phuong Anh Pham-Viernickel, Benjamin Rosner

**Affiliations:** 1 Cleveland Clinic Foundation, Cleveland, OH USA; 2 Ambience Healthcare, San Francisco, CA USA

**Keywords:** Business and industry, Health care, Medical research, Scientific community, Social sciences

## Abstract

Artificial intelligence scribes are increasingly common in clinical settings, but a blueprint for deploying them efficiently at scale is lacking. We share information about the Cleveland Clinic’s vendor-health system deployment partnership consisting of four pillars: governance, training and onboarding, support, and real time learning. This partnership enabled deployment and onboarding of over 4000 ambulatory care clinicians in 4 months and can inform other health systems tasked with rapid enterprise-wide deployment.

## Introduction

Ambient artificial intelligence (AI) scribes and their associated platforms promise to transform healthcare delivery in a way not seen since broad adoption of the electronic health record (EHR) following the 2009 Health Information Technology for Economic and Clinical Health (HITECH) Act^1^. The rapid uptake of ambient AI scribes has largely been driven by demand from clinicians seeking relief from the modern documentation burden epidemic^2^. However, as health systems select AI scribes for use by their workforces, few have described their experiences with how to rapidly deploy a selected product across the enterprise efficiently.

Ambient AI scribes are large language model (LLM)-enabled tools, often integrated with the EHR, that with consent, passively record the patient-clinician conversation at the point of care and leverage LLMs to convert the recording into a formatted, drafted encounter note in the EHR for the clinician to review, edit, and sign^3^. Based on early evidence, the benefits of this technology include subjective improvements in clinician burnout^4–6^, cognitive task load^5,7^, and joy in practice^8^, as well as objective indicators such as increased revenue^9^. Some benefits have been found to be tied to the encounter-level rates of utilization^10^ (e.g. the higher the proportion of eligible encounters for which the tool is used, the greater the benefit). However, “adoption” in some studies is defined according to whether a clinician has ever used the tool rather than the degree to which it is used^11^. With some adoption rates reported to hover around 20%-42%^11^, operational leaders face uncertainty about whether they will realize sufficient utilization to achieve the desired benefits for their workforce.

While sustained usage in every day clinical practice is one consideration that health systems may assess following AI scribe deployment, there are many others including user satisfaction, ease of use, facility of EHR integration, and cost. By some estimates, there are more than 100 commercial vendors of ambient AI scribes today^12^. Although single vendor pilot evaluations are common^13–15^, some leading health systems, including the Cleveland Clinic Foundation (CCF), have undertaken methodical approaches to evaluate multiple ambient AI scribe products in parallel^5,16^. However, after evaluation, few health systems have described successful practices and learnings for rapidly deploying a selected solution at scale that achieves high levels of utilization and satisfaction. In this article, we describe lessons learned from a vendor-health system partnership that enabled successful deployment of an AI scribe solution to over 4000 ambulatory care clinicians in a 4-month time frame.

### Setting

CCF is a large urban academic medical center consisting of 23 hospitals and 276 outpatient locations employing approximately 80,000 caregivers worldwide. Its flagship hospital, headquartered in northeast Ohio, is a quaternary care center that has been recognized in the Best Hospitals Honor Roll of the U.S. News and World Report’s annual rankings^17^. Like many health systems, CCF has faced challenges with clinician burnout, documentation burden, and potentially avoidable clinician attrition. Despite existing operational investment in traditional scribes, CCF leadership recognized the important opportunity that ambient AI scribes offered to address these issues at scale. After conducting its own internal evaluation of AI scribe products^18,19^, CCF partnered with Ambience Healthcare to achieve the shared goals of rapid deployment to over 4000 clinicians within a 4-month period; co-evaluation and ongoing monitoring of value metrics such as utilization, documentation time, clinician satisfaction, and financial indicators; and future partnership opportunities for expansion beyond ambulatory care.

### Rapid Scaling from Pilot to Enterprise Deployment

Following a pilot evaluation, enterprise-wide ambulatory care deployment of the AI scribe began on March 10, 2025. The task of a rapid deployment at enterprise scale involved: (1) Establishing a robust governance structure for a vendor-health system partnership (2) ensuring appropriate training and onboarding, (3) establishing easily accessible support systems, and (4) promoting real-time learning through continuous feedback.

### Governance

Governance of the deployment and rollout to the full ambulatory enterprise was multi-disciplinary in nature and consisted of a vendor-health system partnership to identify and establish cross-functional stakeholder groups including Information Technology (IT), informatics, clinician leadership, and revenue cycle management (RCM).

**Fig. 1:**
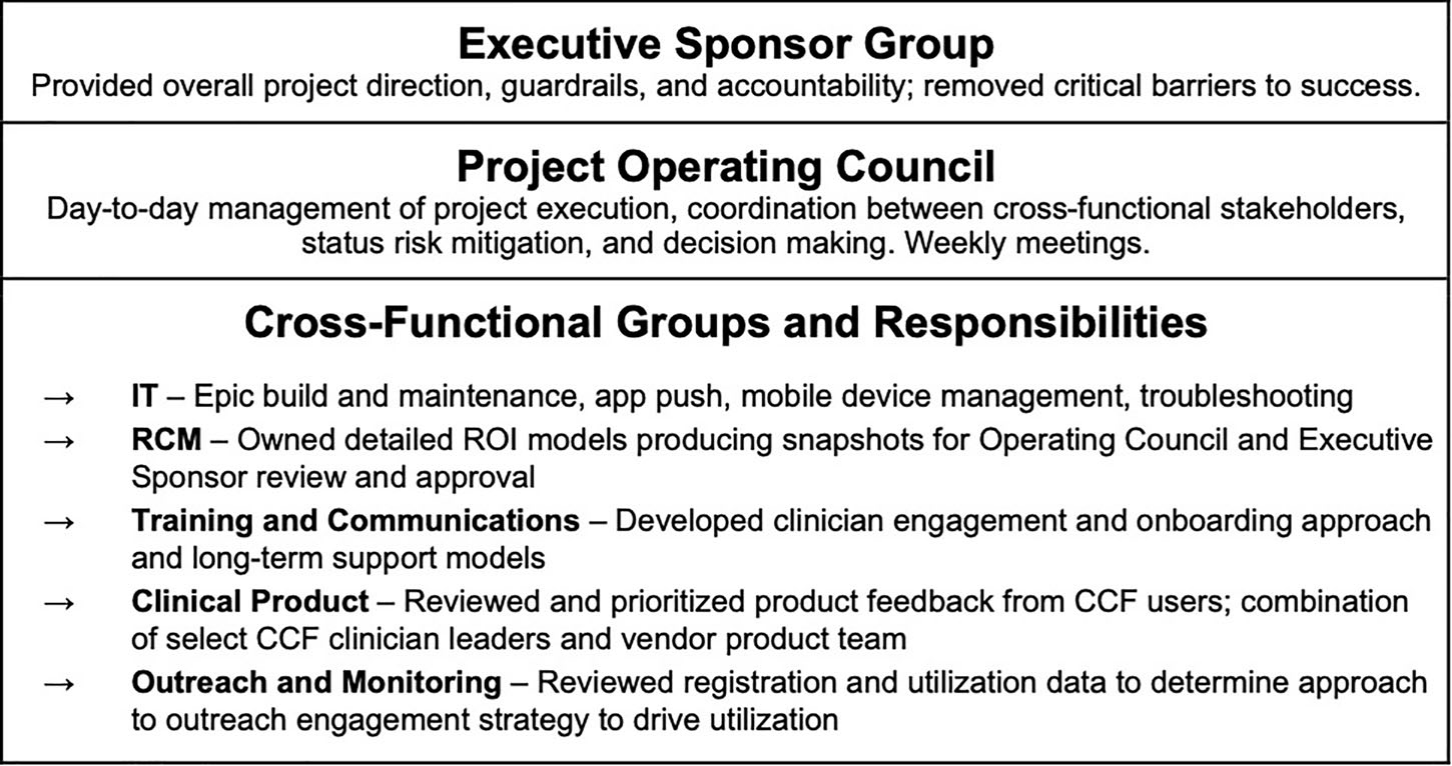
Governance Structure. IT: Information Technology; RCM: Revenue Cycle Management; ROI: Return on Investment; CCF: Cleveland Clinic Foundation.

First, an Executive Sponsor Group was formed and tasked with central oversight to provide direction, guardrails, accountability, and to identify and remove barriers (Fig. 1). A Project Operating Council was charged with on-the-ground management and project execution, coordinating between cross-functional stakeholders and making necessary deployment decisions. Cross-functional health system and vendor-partnered groups consisted of: An IT group for EHR build and maintenance, mobile device management, and troubleshooting; an RCM group to identify and evaluate value indicators; a Training and Communications group to engage clinicians and to develop, communicate, and deliver onboarding and training; a Clinical Product group to review and prioritize user feedback; and an Outreach and Monitoring Group to review utilization and promote adoption.

### Training and Onboarding

To align vendor and health system incentives, contractual milestones were established, consisting of onboarding a predetermined number of clinicians in each of four waves. The first (Wave 0) began with onboarding clinicians who had participated in the pilot using other scribing solutions. This approach minimized the interruption that these users experienced in scribe availability. The subsequent wave consisted of training and onboarding 80% of the total target users, especially those in primary care (Wave 1). Specialties representing 10% of target users were identified for Wave 2, and complex specialties representing the remaining 10% of target users, particularly those in which AI model maturity was being optimized, were identified for Wave 3. (Table 1) To accommodate clinician scheduling needs, each wave remained open for onboarding until the end of the 4-month rollout, helping to mitigate training boluses at the opening of each wave.

**Table 1:** Waterfall analysis of clinician onboarding

Wave	Eligible Clinicians (n)	Clinicians who Attended Training (% of eligible)	Clinicians who Attended Training and Used the AI Scribe 1 or more times (% of trained in each wave)
Pilot	108	104 (96.3%)	104 (100%)
Wave 0	188	186 (98.9%)	165 (88.7%)
Wave 1	4,525	4,193 (92.7%)	3,574 (85.2%)
Wave 2	664	644 (97.0%)	532 (82.6%)
Wave 3	645	621 (96.3%)	451 (72.6%)
Total	6,130	5,748 (93.8%)	4,826 (84.0%)

**Fig. 2:**
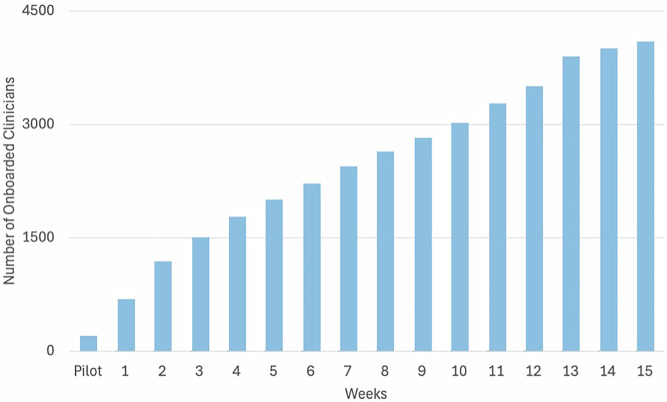
Cumulative number of ambulatory care clinicians onboarded per week during the enterprise-wide deployment.

To ensure rapid uptake, clinician user trainings hosted by the vendor were offered as live virtual group sessions three times daily with adequate time allocated for question and answer. To promote proper usage from the start and to reduce the number of clinicians who might not gain full value from the product, CCF required clinicians to receive this training before being granted access to the tool. All trainings included in-person representation from CCF’s Chief Medical Information Officer (CMIO), Associate CMIOs, or physician superusers. Trainings were held at existing departmental meetings as requested, and self-training modules for those with barriers to live virtual training sessions were integrated into the CCF Learning Management System. Training session registration was managed by the CCF Learning Management System, and attendance was promoted at the departmental level by department leadership who were equipped with communications packages to support cascaded messaging. Over 4000 clinicians were trained and onboarded in just under 16 weeks (Fig. 2).

### Support

To make support as frictionless as possible, the vendor provided direct channels for clinician communication via live mobile chat, staffed with human support personnel 24 hours a day, 7 days a week. A method to facilitate an informed handoff from the CCF Service Desk to the vendor support team was also implemented to efficiently support users who might otherwise be in the habit of directing questions to the CCF Service Desk. A process for reciprocal handoffs from the vendor to the CCF Information Technology Division team was also established for requests that were not related to the ambient tool. During the enterprise roll out, when support for change management is most critical, CCF maintained rapid feedback loops fielding over 900 inquiries with an average response time of two minutes. Most common support inquiries consisted of custom physical exam template requests, multi-clinician workflow configuration settings, requests for draft corrections and content updates, and AI scribe policies at CCF.

### Real Time Learning through Continuous Feedback

To be able to continuously identify friction points, adoption challenges, and other areas requiring attention in real time, CCF and the vendor operationalized multiple feedback channels. Feedback coming through the support channels including, for example, requests to improve specialty-specific output, were prioritized for rapid updates so that timely learning cycles could occur. In addition to feedback captured through the CCF Service Desk and the vendor support team, department chairs were empowered with dedicated dashboards to provide insights into their departments’ own training and onboarding progress, and to identify of areas needing additional support. For example, this process surfaced opportunities to modify training offerings for specialties with prohibitive clinical schedules, including expanding in person session offerings, extra time at departmental meetings, and greater asynchronous offerings once it had been established in a measured way that appropriate training could be achieved asynchronously. We also learned that by empowering department chairs with iterative outreach messaging we could reduce the load on department chairs while driving adoption.

An additional channel for real-time learning included dedicated office hours for users to share efficiency tips learned along the way. A provider advisory group was also tasked with collecting feedback and learnings about specialty-specific content, formatting, features, and settings that proved valuable in making product adjustments and in driving high utilization rates across specialties.

### Key Takeaways

The approach described above enabled CCF to onboard over 4000 clinicians representing approximately 80% of its ambulatory care clinicians within 4 months. Now, more than 12 months post-enterprise-wide deployment, over 4800 CCF clinicians have used the AI scribe across more than 3.5 M encounters.

**Fig. 3:**
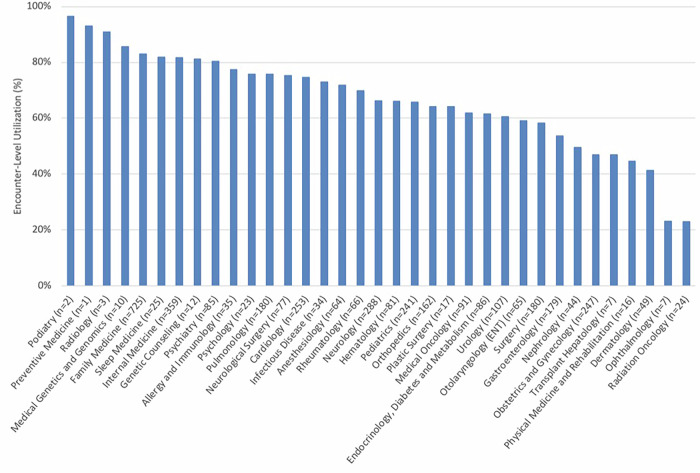
AI scribe 12-month encounter-level utilization by specialty. *n* = number of providers per specialty.

In addition, CCF maintained high utilization and high success standards during the enterprise roll out and beyond. This included, for example, 70% overall encounter-level utilization among established users (those clinicians who have used the scribing tool at least 50 times since deployment. Figure 3) after a full year of deployment. Encounter-level utilization — the proportion of eligible encounters in which a clinician uses the AI scribe — is a foundational metric directly tied to realizing value at scale. It is a more demanding measure than user adoption, which captures only whether a clinician has ever used the tool. In the published literature, adoption rates for AI scribes have been reported in the range of 20–42%^11^, and median utilization (IQR) as high as 52.5% (17.9–81.0%)^20^. Several factors are understood to limit utilization more broadly, including resistance to change, workflow heterogeneity (e.g., clinicians with a predominantly copy-forward workflow), lack of awareness, limited quality and reliability of AI-generated notes, and underlying models that are not optimized for complex specialty use cases. CCF’s rollout plan proactively addressed as many of these underlying factors as possible, including standards set by CCF and the vendor for workflow and content quality, particularly those being developed for specialties.

At enterprise volume, CCF continues to realize durable results including continued clinician satisfaction (Net Promoter Score of 60, CSAT score of 96.6%, and 60.0% of users agreeing or strongly agreeing that the tool has increased their likelihood to remain in practice). These results are outcomes that reflect deliberate organizational choices at each phase of deployment.

### Future Directions

Vendor tools play an important role in the healthcare ecosystem. Just as the quality and workflow compatibility of a given product are essential for its acceptability among end users and healthcare system leaders, so too is a well-designed, governed, and executed deployment. However, a successful vendor-health system partnership rarely concludes at the end of a deployment. Instead, the success of that partnership in the long term may rely just as much on both parties’ ongoing commitments to optimize tools and to innovate in the development of new features. CCF and the vendor continue to do so through monitoring and optimization of the ambulatory product, developing a framework for measuring multiple dimensions of value, partnering on the expansion into the emergency department and inpatient care settings and developing tools to serve other clinicians with unique workflows such as nursing.

While not every health system needs to execute enterprise-wide ambulatory care deployment in such a short timeframe, CCF’s experience provides a framework for health systems seeking to evaluate and implement AI scribes at enterprise scale.

## Data Availability

Data involving healthcare operations of CCF and data such as User Action Log data which may contain protected health information are not publicly available. Other data may be made available by the corresponding author upon reasonable request.
